# Post-Mortem Computed Tomographic Features of the Most Caudal Lumbar Vertebrae, Anatomical Variations and Acquired Osseous Pathological Changes, in a Mixed Population of Horses

**DOI:** 10.3390/ani13040743

**Published:** 2023-02-19

**Authors:** Nicola Scilimati, Giovanni Angeli, Antonio Di Meo, Cecilia Dall’Aglio, Marco Pepe, Francesca Beccati

**Affiliations:** 1 Veterinary Teaching Hospital, Department of Veterinary Medicine, University of Perugia, 06126 Perugia, PG, Italy; 2 Sport Horse Research Centre, Department of Veterinary Medicine, University of Perugia, 06126 Perugia, PG, Italy

**Keywords:** anatomy, bony changes, equine, diagnostic imaging, lumbar region

## Abstract

**Simple Summary:**

Conventional diagnostic techniques cannot always provide a diagnosis for horses with back pain. A better visualization of the vertebral morphologic details can be achieved by using advanced diagnostic imaging tools. For this reason, this study presented a detailed post*-mortem* computed tomographic description of the anatomical variations and acquired osseous pathological changes in the equine caudal lumbar region that have never been reported. The authors hypothesized that body weight, age, previous use, and some anatomical variations might be correlated with the presence and severity of the lesions. The results partially supported the initial hypotheses because the concomitant presence of anatomical variations and acquired osseous pathological changes at the same level, and the correlation between age and the presence of some degenerative pathologies were observed. Furthermore, this study provided additional tomographic information on bone features in both pathological and non-pathological specimens. However, other future studies are needed to correlate the clinical significance of the anatomical variations and acquired osseous pathologies of the equine lumbar vertebrae.

**Abstract:**

The radiographic, ultrasonographic, and scintigraphic findings of horses with thoracolumbosacral pain have been previously reported. In this study, the computed tomographic appearance of anatomical variations and pathological changes of the equine caudal lumbar region through a post-mortem examination were investigated. A total of 40 horses that had died or were submitted for euthanasia, for reasons unrelated to the study, were included in the study. From all the specimens, the modified vertebral system was adopted to evaluate and describe the four most caudal lumbar vertebrae, which were numbered from a caudal reference point (lumbosacral junction), with the segment number designated within parentheses (i.e., L(i)-L(iv)). Contact of the spinous processes was detected in 21 specimens (54%) and fusion in 6 specimens (15%). Lumbar spondylosis was seen in 17 specimens (42.5%), more commonly on the lateral aspect or on both ventral and lateral aspects in 12 specimens (71%). The presence of spondylosis was found more commonly in older horses (*p* < 0.001). There was no difference in bony density in specimens with spondylosis or spinous processes contact compared to specimens without. The highest prevalence of bony changes was found at L(ii)-L(i) intertransverse joints in 28 specimens (97%) on the left and in 22 specimens (96%) on the right side. Spondylolisthesis and partial fusion of the L(ii)-L(i) vertebral disc were found in association with degenerative pathologies. This study showed a high frequency of several anatomical variations and acquired osseous pathological changes in the most caudal lumbar vertebrae via a CT examination.

## 1. Introduction

Several vertebral disorders have been identified as the causes of chronic poor performance in horses [1]. The impingement of spinous processes (SPs) [*Processus spinosus;* i.e., dorsal spinous process], osteoarthritis of the intertransverse joints (ITJs) [*Articulationes intertransversariae lumbales]* and articular processes joints (APJs) [*Articulationes processuum articularium]*, ankylosis of the ITJs, vertebral body osteophytes, and degenerative disc disease are only some of the osseous pathologies known to develop in the vertebral column [2,3]. Furthermore, the morphological variations in the lumbosacral area, such as fusion of the lumbar SPs and transverse processes, presence of lumbosacral transitional vertebrae, and varying numbers of lumbar and sacral vertebrae, have been previously described [2,4,5,6]. Some variations in the morphology of the thoracolumbar vertebral bodies, SPs, and joints are known to occur [4]. Being aware of the incidence of vertebral anomalies is essential to distinguish between acquired pathological changes and functional anatomic variations [4].

The clinical inability to localize vertebral abnormalities or the factors contributing to soreness result in unresolved spine problems [3]. Advanced diagnostic imaging techniques allow a better visualization of vertebral and pelvic morphologic details than the first-level diagnostic techniques, enhancing the knowledge of the anatomical variants and pathologies in the equine vertebral column [7]. Computed tomography (CT) and magnetic resonance imaging (MRI) are increasingly being applied in horses with suspected cervical diseases [8,9,10,11]. However, in vivo CT evaluation of the lumbar area is not applicable in adult horses due to size limitations [12]. Recently, post-mortem CT has been used to examine the osseous variations in the equine cervical vertebral column [8] and the osseous anatomy of the axial skeleton from the caudal cervical area up to the sacrococcygeal region in horses [13], and also to evaluate the presence of the ITJs in young foals [14].

This study was performed to assess the features and morphological appearance of the anatomical variations and acquired osseous pathologies in the caudal lumbar vertebral column in a mixed population (i.e., variable breeds, ages, sex, body weight, and discipline) of horses that died or were euthanized for reasons unrelated to this study via a post-mortem CT examination performed on specimens.

It was hypothesized that age, body weight, and some anatomical variations might be correlated with the presence and severity of the osseous pathologies detected. It was also hypothesized that specimens with contact or fusion of the SPs and vertebral body spondylosis might present with higher bone density at that level.

## 2. Materials and Methods

### 2.1. Horses

No specific authorization from an Animal Ethics Committee was required for this study. Forty horses aged ≥ 4 years that died or were euthanized at the Veterinary Teaching Hospital of the University of Perugia (Italy) between November 2019 and April 2022 for reasons unrelated to this study (i.e., colic (*n* = 34), fracture (*n* = 3), neoplasia (*n* = 2), bilateral blindness (*n* = 1)), were included in the study following signed owner consent. Anatomical variations and acquired bony changes detected at post-mortem examination by gross inspection of bone specimens of 31 horses were presented in a previous paper [6]. The age, breed, body weight, sex (male or female), and previous use of the horses were recorded. At admission, the age of the horses was determined by their passport, and the body weight by a calibrated weighbridge. The breeds consisted of Warmblood (*n* = 17), Arab (*n* = 5), Anglo-Arabian (*n* = 4), Thoroughbred (*n* = 4), Standardbred (*n* = 3), and others (*n* = 7). The study included 27 female and 13 male horses, ranging in age between 5 and 27 years (mean: 16 years; standard deviation: 6 years) and weighing between 400 and 620 kg (mean: 501 kg; standard deviation: 63.2 kg) (Table 1).

After necropsy, the lumbosacroiliac region, including the four most caudal lumbar vertebrae, was removed intact from each carcass. The ilial wings were isolated from the pelvis by bilateral cutting of the neck of each ilium, close to the acetabulum. The modified vertebral system referenced from the lumbosacral junction (i.e., L(i), L(ii), L(iii), L(iv)), previously used by Haussler and colleagues [3,4], was adopted. This reference system allowed the designation of the lumbar vertebrae, because the total number of lumbar vertebrae for each horse was unknown. All the specimens underwent a CT examination before being dissected. Only specimens scanned within 24 h after death or euthanasia were included in the study.

### 2.2. CT Protocol

The spines were scanned, mimicking the horses in a standing position in a custom-designed CT tray. A 16-slice CT scanner (Fujifilm-FCT-Speedia HD, Duesseldorf, Germany) with a voltage of 120 kV, current of 100 mA, and tube rotation time of 1 s was used. Helical acquisition (pitch 0.8) was performed using a 512 × 512 matrix with a field of view of 50 cm, an effective slice width of 3 mm, and collimated slice width of 1.25 mm.

### 2.3. CT Evaluation

The CT images were available in the Digital Imaging and Communications in Medicine (DICOM) format and then transferred to a workstation and reformatting software (Horos) to produce the reformatted images. All the images were successively evaluated by two operators concurrently and together, one (GA) with more than 20 years of experience in CT image interpretation and one PhD student (NS), and then a consensus was reached. The images were reconstructed in 3D multiplanar rendering, surface rendering, and volume rendering. Multiplanar reformatting images were used to obtain 1.5-mm-thick slices in the dorsal, transverse, and sagittal planes using a sharp (12) kernel and displayed in bone and soft tissue windows. 

The normal CT appearance of the SPs, APJs, ITJs, and vertebral bodies was described using, as a reference, two specimens (specimens 5 and 36) without pathological changes or anatomical variations of the caudal lumbar region evaluated during the anatomical dissection performed in our previous study [6]. All the specimens were examined for the presence of anatomical variations (fusion of the SPs, fusion of the L(ii)-L(i) intervertebral joints), designated as congenital or developmental abnormalities of the bony structures [14], and acquired pathological changes (contact of the SPs, osseous pathological changes of the ITJs and APJs, spondylosis, spondylolisthesis) in the caudal lumbar region (four most caudal lumbar vertebrae). The presence of a visible joint space between ITJs, APJs, and vertebral bodies was considered normal. Ankylosis of the APJs was defined as a pathological acquired condition leading to a partial or complete absence of the joint space. Otherwise, the partial or complete absence of joint space between the ITJs and vertebral bodies was defined as fusion without making any distinction between congenital or acquired fusion (ankylosis), regardless of whether or not bone remodeling/new bone formation was detected [13]. The changes detected in the most caudal lumbar spine, as well as the alignment of the vertebral bodies and the changes in the size of the intervertebral discs, were recorded as follows. The SPs of the vertebrae were evaluated for the presence of contact on the top with measurement of the relative mean Hounsfield units (HU) or for the presence of fusion recording the exact localization and the mean HU.

The presence of the ITJs between the transverse processes of the four most caudal lumbar vertebrae was recorded. The ITJs were evaluated for their localization, laterality (left, right, or bilateral), and symmetry. For each specimen, the length of every ITJ was measured regardless of whether the ITJs were fused or not, drawing a line along the ITJ space from the axial to the abaxial extremity on the not fused ITJs, or at the level of an imaginary joint space on the fused ITJs. A circle with a diameter equal to the length of the respective lumbar ITJ was drawn. The periarticular bone surface of both fused and not fused ITJs within the circle was then evaluated for mean, minimum and maximum HU, and standard deviation. Furthermore, the length of every lumbar ITJ was then divided into three sections (axial, middle, and abaxial parts). The length of each part, equal to one-third of the length of each ITJ, was used as the diameter of the respective drawn circles, within which the mean HU of the periarticular bone surface of both fused and not fused ITJs at the level of the cranial and caudal lumbar transverse processes was calculated. 

The articular margins and joint spaces of the ITJs and APJs were evaluated for the presence and severity of osseous pathological changes as evidence of new bone proliferation (i.e., osteophytes and/or enthesophytes).

The severity of pathological changes of the joint surface and the change in the size of the ITJs and APJs were scored using the readapted grading system adopted in our recent anatomical study about the lumbosacroiliac region [6]: Grade 0 = mild, bony changes affecting < 25% of the articular or periarticular surfaces; Grade 1 = moderate, bony changes affecting 25%–50% of the articular or periarticular surfaces; and Grade 2 = severe, bony changes affecting > 50% of the articular or periarticular surfaces. Furthermore, Grade 3 = ankylosis, bone proliferation with the bridging or obliteration of the periarticular surfaces was added to the grading system for evaluating bony changes involving only the APJs. 

The mean HU of the cranial and caudal vertebral endplates of the vertebral bodies was recorded, from the cranial endplate of L(iv) to the cranial endplate of L(i). If present, vertebral spondylosis and its localization were recorded. Spondylosis was classified as the presence of new bone formation involving the vertebral bodies [3]. The presence of displacement of one or more vertebrae (spondylolisthesis) was recorded [15] and the definitions of ‘anterolisthesis’ and ‘retrolisthesis’ normally used in humans [16], were readapted for veterinary use. Dorsal spondylolisthesis describes the dorsal displacement of a vertebra relative to the horizontal orientation of the vertebral column, whereas ventral spondylolisthesis describes the ventral displacement of a vertebra relative to the normal vertebral axis. Furthermore, the presence of a narrowed or fused L(ii)-L(i) intervertebral disc, previously reported in ultrasonographic and scintigraphic studies [17,18], with or without concurrent lumbar ITJ fusion or APJ ankylosis, was recorded. 

### 2.4. Data Analysis

The data were analyzed using commercially available statistical software (JASP Team 2022, version 0.16.1). Male horses and geldings were pooled together for the statistical analysis. The categorical data included sex (male vs. female), breed (Warmblood, Thoroughbred, Standardbred, Arab, Anglo-Arabian, and other breeds), and previous use (jumping, race, show, pleasure, and others). A descriptive statistical analysis was applied for specimens’ data (age, sex, body weight, breed), anatomical variations (presence of the ITJs), osseous pathological changes (contact between the lumbar SPs, arthropathy of the ITJs, APJs, and spondylosis), and mean HU of the SPs, ITJs, and vertebral bodies. The continuous data (age, body weight, and mean HU measurement) were tested for normality using the Shapiro-Wilk test and for homoscedasticity using Levene’s test, and statistical tests were performed as appropriate. The Student’s *t*-test or Mann-Whitney U test was used, as appropriate, to test for the differences between fusion or contact of the SPs (no vs. yes), presence of the ITJs at L(iii)-L(ii) (no vs. yes), a fusion of the ITJs at each site, including L(ii)-L(i) (no vs. yes), osseous pathological changes at each ITJ (no vs. yes) and APJ (no vs. yes), presence of spondylosis at each site (no vs. yes), and age and body weight.

The Student’s *t*-test or Mann-Whitney U test was used, as appropriate, to test for the differences in the length of each ITJ and mean HU of the vertebral bodies between sexes. Analysis of the variance or the Kruskal-Wallis test, as appropriate, was used to test the differences in the length of the ITJs and mean HU of the vertebral bodies, as well as the grades of the pathological changes of the ITJs, and age and body weight. Post hoc analysis was performed using Tukey’s or Dunn’s test as appropriate. Pearson’s *r* coefficient was calculated to examine the correlation between age, body weight, and mean HU of the SP fusion and contact, length of each ITJs, mean HU of the ITJs, and mean HU of the vertebral bodies. The Student’s *t*-test or Mann-Whitney U test was used, as appropriate, to test for the differences between the length of the ITJs and mean HU of the ITJs and the presence of fusion of the ITJs; and between the mean HU of the vertebral bodies and presence of spondylosis at each site. The chi-square or Fisher’s exact tests were used, as appropriate, for examining the differences in the degree of pathological changes of the APJs, degree of pathological changes, and fusion of the ITJs and spondylosis. *P*-value < 0.05 indicated a statistically significant difference.

## 3. Results

### 3.1. Horses

Thirty-one horses had been in athletic use; reported athletic uses included jumping (*n* = 17), racing (*n* = 7), showing (*n* = 4), and others (*n* = 3). The remaining nonathletic use horses included in the study were pleasure or breeding horses (*n* = 9) (Table 1).

### 3.2. CT Findings

**Figure 1:**
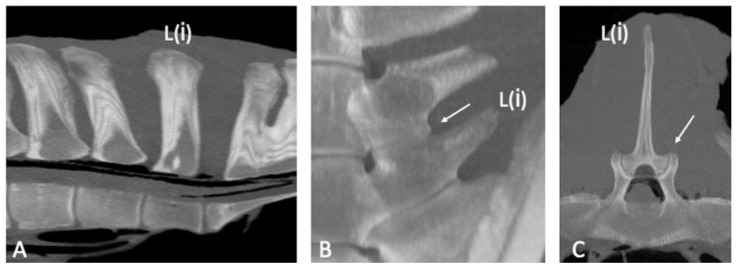
Sagittal ((**A**); cranial is toward the left), dorsal ((**B**); cranial is toward the top) and transverse ((**C**); dorsal is toward the top) multiplanar reconstruction computed tomographic images of the lumbar specimen 5, using maximum intensity projection. (**A**) Presence of visible intervertebral space and absence of spondylosis, spondylolisthesis and lumbar spinous processes contact or fusion. Note the spinous process (SP) of L(i) perpendicular to the orientation of the column axis, mild irregularity of the bone surface of the L(ii) SP onto the dorsocranial aspect and contact between the S1 and S2 SPs and moderate irregularity of the dorsocaudal aspect of the S1 SP and dorsocranial aspect of the S2 SP; fusion of the ventral portion and part of the middle portion of the S1-S2 SPs (**B**,**C**) Regularity of the articular margins and the joint space of the L(ii)-L(i) intertransverse joint and L(ii)-L(i) articular processes joint (arrow); the L(ii)-L(i) intertransverse space is mildly narrowed on the abaxial aspect. L(i): the most caudal lumbar vertebra.

The CT appearance of two specimens without abnormalities [6] was characterized by homogeneous bone density (HU) of the bony structures (SPs, APJs, ITJs, and vertebral bodies). Differences in bone density between cortical (>1000 HU) and trabecular bone (>300 HU) of the vertebral structures were observed [19]. The periarticular bone margins were regular, and well-defined joint spaces were detected (Figure 1).

**Figure 2:**
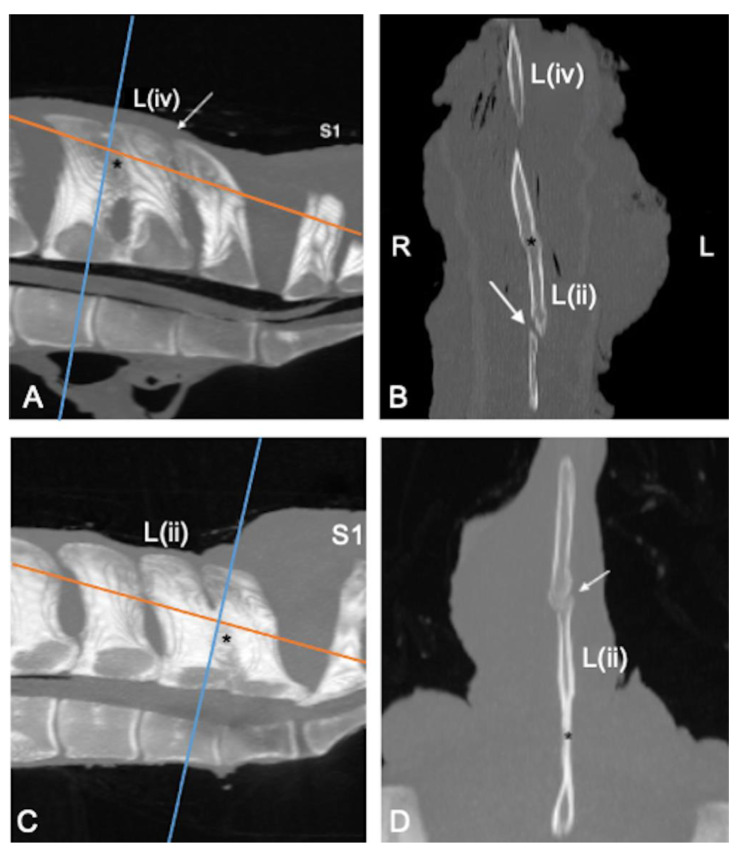
Sagittal (**A**,**C**); cranial is toward the left and dorsal (**B**,**D**); cranial is toward the top multiplanar reconstruction computed tomographic images of the lumbosacral specimens 8 and 25, using maximum intensity projection (MIP). (**A**,**B**) Complete obliteration of the L(iii)-L(ii) interspinous space due to fusion of the dorsal and middle portion of the respective spinous processes (SPs) (asterisk) and severe narrowing of the L(ii)-L(i) interspinous space (arrow). (**C**,**D**) Fusion of the middle and ventral portion of the L(ii)-L(i) SPs (asterisk). There is contact on the dorsal and minimally on the middle portion of the L(iii)-L(ii) SPs with reduced density of the bone and lack of compact bone (arrow); the contact is difficult to be seen on sagittal MIP image (**C**) but clearly highlighted on the dorsal image (**D**), which show also new bone formation, abaxially. (**B**,**D**) Note the absence of increased bone density on the spongious bone surrounding contact or fusion of the SPs. L: left; R: right; L(iv): fourth to the most caudal lumbar vertebra; L(ii): second to the most caudal lumbar vertebra; S1: first sacral vertebra.

Contact of the SPs was noticed in 21 specimens (54%), and fusion was found in 6 specimens (15%) (Figure 2). The details of location and mean HU are presented in Table 2.

**Figure 3:**
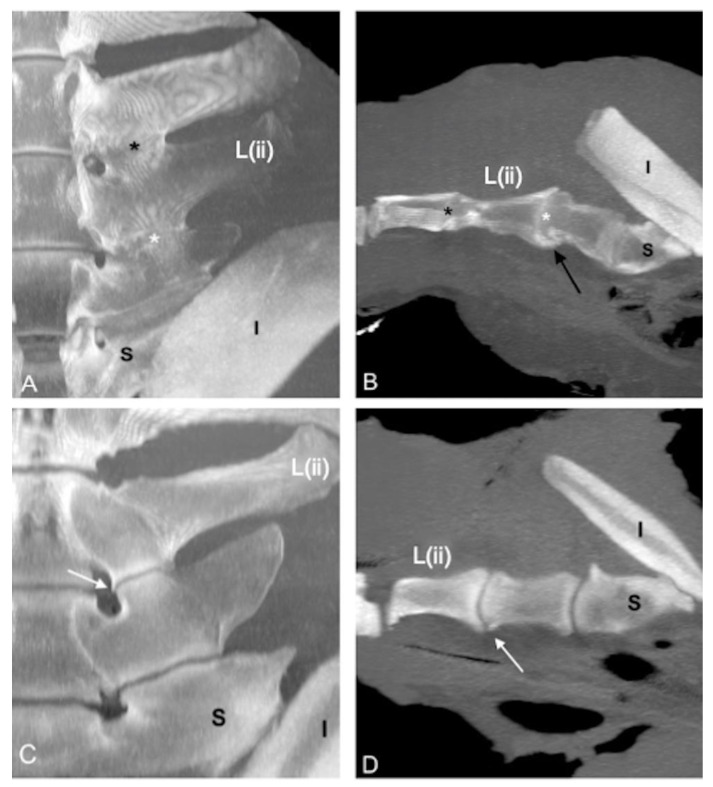
Dorsal ((**A**,**C**); cranial is toward the top) and parasagittal ((**B**,**D**); cranial is toward the left) multiplanar reconstruction computed tomographic images of the lumbosacroiliac specimens 43 and 9, using maximum intensity projection (MIP). (**A**,**B**) Absence of joint space between the left L(iii)-L(ii) (black asterisk) and L(ii)-L(i) intertransverse joints (ITJs) (white asterisk) and periarticular bone formation onto the ventral margin of L(ii)-L(i) ITJ with complete bridging of the joint (black arrow). (**C**,**D**) Visible joint space between the left L(ii)-L(i) ITJ with the presence of a small osteophyte involving the ventral periarticular margin of L(i) (white arrow). L: left; R: right; L(ii): second to the most caudal lumbar vertebra; I: ilium; S: sacrum.

**Figure 4:**
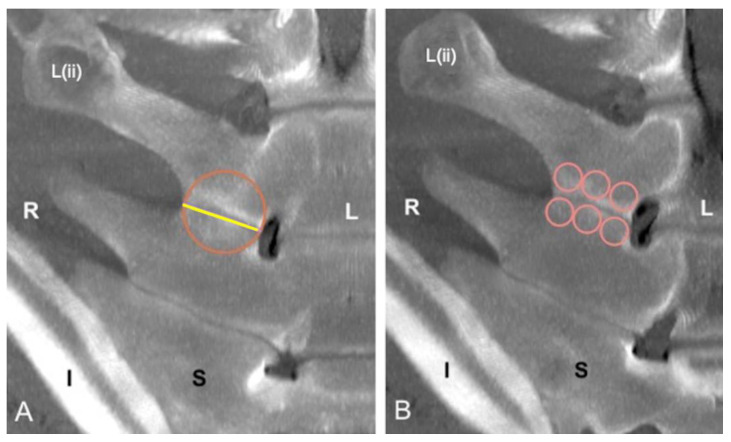
Dorsal multiplanar reconstruction computed tomographic images of the lumbosacroiliac specimen 40; cranial is toward the top of the images. (**A**) Measurement of the length of the right L(ii)-L(i) intertransverse joint (ITJ) and assessment of mean, minimum, and maximum Hounsfield unit (HU) and standard deviation of the periarticular surface by drawing a circle with a diameter equal to the length of the respective ITJ. (**B**) The right intertransverse periarticular bone surface of L(ii) and L(i) was divided into three sections (axial, middle, and abaxial part), and their respective mean HU was calculated by drawing on each part a small circle, with a diameter equal to one-third of the length of each ITJ. L: left; R: right; L(ii): second to the most caudal lumbar vertebra I: ilium; S: sacrum.

The L(iv)-L(iii) ITJ was found in 1 specimen (2%) on the right side (length: 2.4 cm; mean HU: 665), and no bony changes were found at that level. The presence and symmetry, mean length, mean HU, fusion, and grade of pathological changes of L(iii)-L(ii) and L(ii)-L(i) ITJs (Figure 3 and Figure 4) are summarized in Table 3.

Horses with L(iii)-L(ii) ITJs had a greater body weight than those without (*p* = 0.008). A positive correlation was found between the left and right lengths (*r* = 0.9) and the mean HU (*r* = 0.73) of the L(iii)-L(ii) ITJs (both *p* < 0.001). Furthermore, a negative correlation between L(iii)-L(ii) and L(ii)-L(i) ITJs mean HU and length (*p* < 0.001) was seen on both left (L(iii)-L(ii): r = −0.81; L(ii)-L(i): r = −0.81) and right side (L(iii)-L(ii): r = −0.71; L(ii)-L(i): r = −0.76).

**Figure 5:**
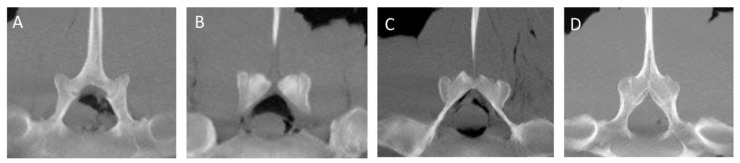
Transverse multiplanar reconstruction computed tomographic images (**A**–**D**) of the lumbar specimens 12, 23, 34, and 25, using maximum intensity projection (MIP); the dorsal is toward the top of the images. There are bilateral mild (**A**), moderate (**B**), and severe (**C**) periarticular osseous changes involving the cranial aspect of the L(ii)-L(i) articular processes joints (APJs). (**D**) There is also complete obliteration of the APJs joint space (ankylosis). L(ii): second to the most caudal lumbar vertebra; L(i): the most caudal lumbar vertebra.

There was a correlation between both the left and right lengths (*r* = 0.6) of the L(ii)-L(i) ITJs and mean bone density (HU) of the adjacent bone on the left and right sides (*r* = 0.7) (both *p* < 0.001). The detected bony changes and their symmetry in the APJs from L(iv)-L(iii) to L(ii)-L(i) (Figure 5) are summarized in Table 4.

**Figure 6:**
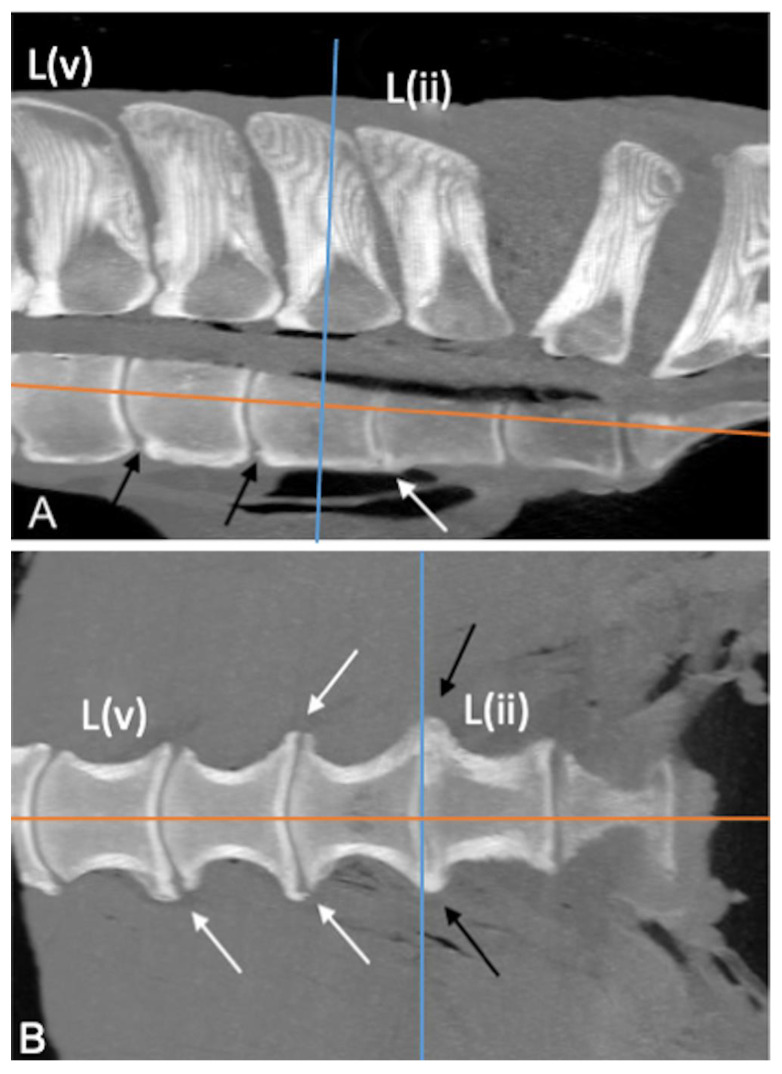
Sagittal (**A**) and dorsal (**B**) multiplanar reconstruction CT images of the lumbar specimen 16; cranial is toward the left. (**A**) Presence of small amounts of new bone formation involving the ventrocranial aspect of the L(iv) and L(iii) vertebral bodies (black arrows) and severe new bone formation causing vertebral bridging between the ventrocaudal aspect of L(iii) and the ventrocranial aspect of L(ii) vertebral bodies (white arrow); note the uncommon divergence of the spinous processes between L(ii) and L(i). (**B**) New bone formation of small/moderate size located at the lateral aspect of the vertebral bodies from L(v) to L(iii) (white arrows). Severe new bone formation onto the lateral aspect of the L(iii)-L(ii) vertebral bodies bilaterally, resulting in fusion of the respective vertebrae (black arrows). L: left; R: right; L(v): fifth to most caudal lumbar vertebra; L(iv): fourth to most caudal lumbar vertebra; L(iii): third to most caudal lumbar vertebra; L(ii): second to most caudal lumbar vertebra; L(i): most caudal lumbar vertebra.

Lumbar spondylosis was detected in 17 specimens (42.5%) and was encountered most on the caudal aspect of L(iv) (10/40) or the cranial aspect of L(iii) (10/40). The new bone formation was noted only on the ventral aspect of the lumbar vertebra(ae) in 5 specimens (29%) and on the lateral aspect or on both ventral and lateral aspects in 12 specimens (71%) (Figure 6).

The details of the mean HU of the vertebral body endplates and localization of spondylosis are summarized in Table 5.

Spondylosis was seen more commonly in specimens from older horses than younger horses (*p* < 0.001). Furthermore, it was more commonly found in specimens with higher grades of osseous changes of the APJs at the level of L(iv)-L(iii) (*p* < 0.05) and L(iii)-L(ii) (*p* < 0.04).

A correlation was observed between the mean HU of caudal L(iv) and L(iii) (r = 0.6; *p* < 0.001), caudal L(iii) and L(ii) (r = 0.66; *p* < 0.001), and cranial L(iii) and L(ii) (r = 0.72; *p* < 0.001), and cranial L(ii) and L(i) (r = 0.5; *p* < 0.001).

A reduction in the mean HU value of the cranial aspect of the vertebral body endplate was observed from L(iv) to L(i) and of the caudal aspect from L(iv) to L(ii). Furthermore, the mean HU value of the caudal aspect (L(iii)-L(i)) was higher than that of the cranial aspect (Table 4).

**Figure 7:**
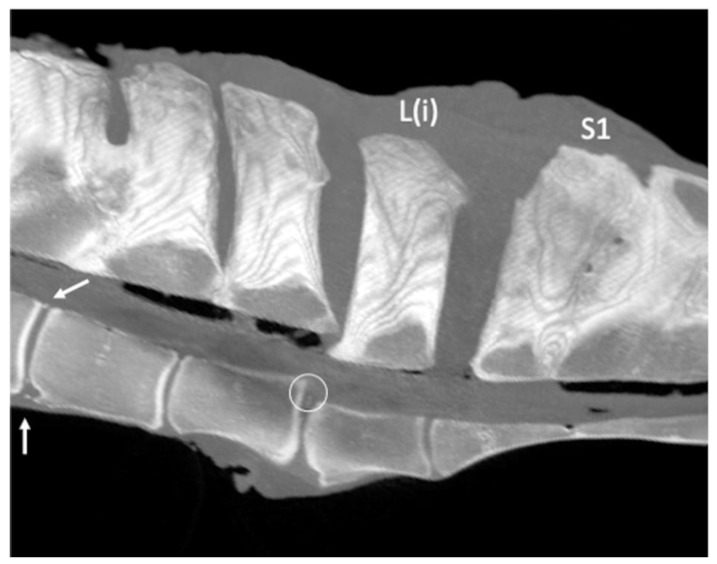
Sagittal multiplanar reconstruction computed tomographic image of the lumbosacroiliac specimen 1 using maximum intensity projection (MIP) (cranial is toward the left) showing the presence of a ventral displacement of L(i) and sacrum relative to the more cranial lumbar vertebrae (ventral spondylolisthesis). Note two small hyperattenuating structures at the level of the dorsal portion of the intervertebral disc (circle), L(iv)-L(iii) intervertebral osteophytes (white arrows) on both the dorsal and ventral aspects, and L(iv)-L(iii) spinous processes (SPs) fusion, as well as a fusion of the sacral SPs. L(iv): fourth to the most caudal lumbar vertebra; L(iii): third to the most caudal lumbar vertebra; L(ii): second to the most caudal lumbar vertebra; L(i): the most caudal lumbar vertebra; S1: the first sacral vertebra.

Ventral spondylolisthesis involving L(i) and the sacrum was found in one specimen, concurrently with a marked change in the L(ii)-L(i) disc and the presence of two small circular hyperattenuating structures attributable to disc mineralization (Figure 7).

Spondylolisthesis was observed in concomitance with bilateral severe osseous changes of the L(ii)-L(i) ITJs and APJs.

**Figure 8:**
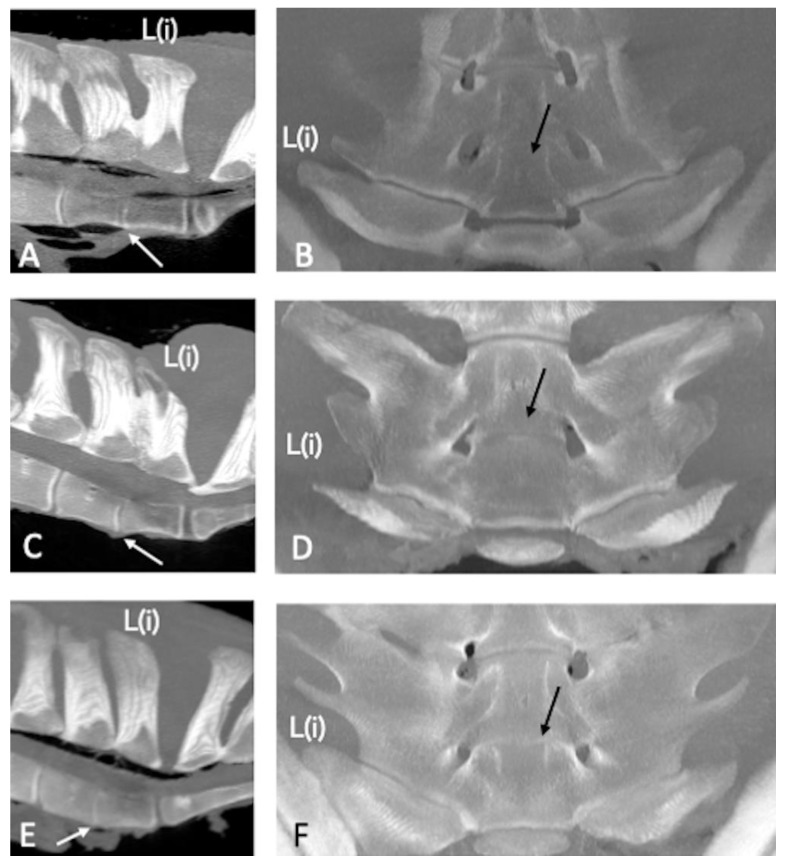
Sagittal (**A**,**C**,**E**); cranial is toward the left) and dorsal (**B**,**D**,**F**); cranial is toward the top) multiplanar reconstruction CT images of the lumbosacroiliac specimens 19, 25, and 33 using maximum intensity projection. (**A**,**C**,**E**) Note the marked narrowing of the L(ii)-L(i) intervertebral discs space (white arrows) with no visible disc space dorsally (dorsal fusion) in comparison with the L(iii)-L(ii) ones, associated with partial fusion of the respective spinous processes (SPs) in specimens 19 and 29. (**B**,**D**,**F**) The L(ii)-L(i) intervertebral disc space is not seen on the ventral aspect (black arrows) in concomitance with bilateral L(ii)-L(i) intertransverse joints (ITJs) fusion in all 3 specimens. L(iii): third to most caudal lumbar vertebra; L(ii): second to most caudal lumbar vertebra; L(i): most caudal lumbar vertebra.

A partial intervertebral joint fusion (dorsal fusion) of L(ii)-L(i) was found in nine specimens (22.5%) (Figure 8).

The partial intervertebral joint fusion of L(ii)-L(i) was detected in concomitance with the bilateral fusion of L(ii)-L(i) ITJs in five specimens (62.5%) and unilateral fusion of L(ii)-L(i) ITJs (on the right side) in one specimen (12.5%). In one spine (12.5%), a partial intervertebral joint fusion of L(ii)-L(i) was detected in association with a fusion of L(iii)-L(ii) ITJs, a fusion of L(ii)-L(i) ITJs, and ankylosis of L(iii)-L(ii) APJs; in another spine (12.5%), the partial intervertebral joint fusion of L(ii)-L(i) was found together with the fusion of L(ii)-L(i) ITJs and ankylosed L(iii)-L(ii) APJs.

## 4. Discussion

This observational post-mortem CT study added information about the prevalence of the osseous pathological changes and anatomical variations in the equine caudal lumbar region from a sample of horses with different age, sex, breed, and previous use. The results partially supported the initial hypotheses because the simultaneous presence of some anatomical variations and of acquired pathological changes at the same level (i.e., the fusion of the L(ii)-L(i) intervertebral joint in concomitance with L(iii)-L(ii) and L(ii)-L(i) ITJs fusion and ankylosis of the L(iii)-L(ii) APJs) was found, and difference in the age among the presence of degenerative pathologies, such as APJ osseous changes and vertebral spondylosis was detected.

In this study, spondylosis was detected in 42.5% of the specimens, more commonly on both the ventral and lateral aspects of the vertebral bodies, without evidence of increased bone density at that level, contrary to our initial hypothesis. Spondylosis is a degenerative process affecting the vertebral body axis, resulting in new bone formation involving adjacent vertebral bodies [15]. Vertebral spondylosis in horses has been reported more frequently, affecting the thoracic spine and, occasionally, the lumbar vertebrae [2,20,21]. These degenerative changes were found especially in the mid-caudal thoracic region (T11–T13), with a low prevalence (2.7–3.4%) in a mixed population of horses with spine pain that underwent radiographic examination of the back. However, lateral-lateral two-dimensional images provided an incomplete overview of the vertebral bodies [1]. Spondylosis of the thoracolumbar region was found in 36% of specimens in a cadaveric study from horses of different breeds considered functionally normal during a clinical examination. At the anatomical dissection, spondylosis was most commonly seen from T11–T13 and at L3 involving the ventrolateral aspect of the vertebral bodies [2]. In the present study, spondylosis was mostly observed in older horses than younger horses. Further studies are needed to determine if there is an association between age and spondylosis. However, this pathological condition was observed more commonly in older horses (>7 years) than horses <7 years of age in previous studies [1,2]. It was suggested that these pathological abnormalities were the result of the repeated strain of the intervertebral discs over time or as a consequence of aging degeneration [1,2]. Potentially, mechanical stress at the attachment of the ventral longitudinal ligament and/or the most peripheral fibers of the intervertebral disc can lead to osteophytes or enthesophytes formation at this level. However, further post-mortem studies are needed [15]. As a result, partial or complete ankylosis of the affected vertebral bodies can occur [15]. In the current study, these findings could explain why authors found no increased bony density of the vertebral body endplates in specimens with spondylosis, since new bone formation involves other structures than the vertebral body endplates. Moreover, osteophytes involving the lumbar vertebrae in horses were more commonly detected on the lateral aspect of the vertebral bodies instead of ventrally as in the thoracic area [15]. In this study, the three-dimensional CT evaluation allowed more accurate identification of these lesions than a standard radiographic examination, avoiding superimposition of the adjacent structures and helping identify lesions circumferentially [9].

In the present study, spondylolisthesis was found only in one specimen at L(i), together with L(ii)-L(i) intervertebral disc modification. This intervertebral malalignment was identified with transrectal ultrasound at the level of L5-L6 or L6-S1 [22], during radiographic examination in the cervical area [23,24], and at the cervicothoracic junction [24] in a mixed population of adult horses. In our study, it was supposed that the L(i) ventral spondylolisthesis, as well as the bilateral severe pathological changes of L(ii)-L(i) ITJs and APJs, might be acquired modifications that developed secondary to the presence of a congenital incomplete L(i) sacralization identified in this specimen [6]. Sacralization is defined as a congenital fusion of the vertebral bodies and the transverse processes of the most caudal lumbar vertebra and the sacral wings of the first sacral vertebra; the latter can be fused unilaterally (incomplete) or bilaterally (complete) [4].

In the equine spine, pathological changes of the APJs most often affect the caudal thoracic and cranial lumbar vertebrae [7]. In the present study, variable degrees of APJ pathological changes were observed commonly from L(iii) to L(i) with high symmetry. This is interesting because the vertebral motility of the caudal lumbar region is normally restricted, and this area is considered less subject to biomechanical stresses than the lumbosacral junction and the thoracic cranial region [25,26]. CT images allowed a better evaluation of the APJ space together with the exact localization and symmetry of the osseous changes in the lumbar region. In our study, no difference was found in age or body weight for APJ pathological changes in accordance with a previous study [7]. However, in another study of racehorses and non-racehorses, the presence and severity of the APJ pathological changes increased with age [20]. In our study, the prevalence of the pathological changes involving the most caudal lumbar vertebrae was found with a higher percentage (70%) than in a recent post-mortem study (54%) [27]. It could be justified by the different methodologies used. The tomographic examination also allows an evaluation of the inner portion of the bone compared to the evaluation at the dissection where immature osteophytes may be damaged by boiling and soaking. However, in the aforementioned study, osseous changes of the caudal lumbar APJs and ITJs were seen more commonly in horses with confirmed lumbosacral region pain compared with a control group, and it was therefore suggested that these pathological findings might contribute to pain [27]. Furthermore, it was hypothesized that abnormalities of the lumbar joints, among which fusion of the L5-L6 ITJs and ankylosis of the L5-L6 APJs, may alter biomechanics, thus the distribution of propulsive forces from the sacroiliac joints to the thoracolumbar vertebral column [27].

CT examination of fused ITJs revealed the presence of uniform trabecular bone with no evidence of periarticular space along its length. Furthermore, a marked difference into the irregularity of the cortical bone surface was not observed between some fused ITJs, which had been easily differentiated in fused and ankylosed during the anatomical dissection in our previous study [6]. This was probably due to the fact that the fusion of the ITJs was also recognized as a developmentally acquired process to increase the stability of the equine lumbar spine [2,3,28], besides the congenital type found in a very young Warmblood foal [14]. In adult horses, the difference between the congenital and developmental ITJ types cannot be easily defined unless a severe new bone formation of the respective periarticular surfaces is detected. For this reason, ITJs without visible joint space were classified as ankylosed, meaning fused, in a recent CT study, regardless of whether it was a congenital or acquired abnormality [13].

In this study, no correlation was found between lumbar spondylosis, ankylosis of the APJs, and fusion of the ITJs, in agreement with previous findings [3,7,21].

In our study, a fusion of the L(ii)-L(ii) intervertebral joint was seen with a similar percentage to Boado and colleagues, although fusion of the L5-L6 was detected ultrasonographically together with a congenital fusion of the lumbosacral intervertebral joint in horses showing lumbosacral region pain [29]. 

Contrary to our hypothesis, the authors did not find higher bone density values in specimens with contact or fusion of the SPs. It was hypothesized that the lack of the difference in bone density of these SPs with contact or fusion was due to lucent zone formation in the compact and spongious bones in concomitance with periosteal reactivity [30] that offset the overall bone density.

The length and mean HU of the L(iii)-L(ii) and L(ii)-L(i) ITJs were strongly correlated. No studies to date involved the measurement of the length of lumbar ITJs. Gorgas and colleagues (2007) found high variability in the length of L6 and the lumbosacral joint space in a radiographic evaluation of the anatomy of the sacroiliac region, supposing that the measurement of these structures by ventrodorsal radiographic images could be inaccurate as a result of the superimposition of the adjacent structures [31]. For this reason, further CT studies would be necessary to assess variability in the length of L6 and the lumbosacral joint space.

This study has some limitations. The main limitation is related to the incomplete isolation of the whole lumbar tract. For this reason, the four most caudal lumbar vertebrae were counted from caudally to cranially, according to the modified vertebral reference system. Accordingly, the present study did not allow for achieving a complete description of the lumbar region because the authors could not identify potential variations in the total number of vertebrae. In addition, the CT findings could not be related to the orthopedic status of the horses, although this was not the aim of the current study.

## 5. Conclusions

In conclusion, this study showed a high frequency of anatomical variations and pathological changes in the most caudal lumbar vertebrae, which have not previously been assessed via a CT examination. This specific region is usually evaluated with more difficulty in vivo by radiographic and transcutaneous ultrasound examinations due to the deep location of the caudal lumbar vertebrae and overlapping of the ilial wings and surrounding muscles. Avant-garde technologies, such as the standing CT system, may allow in the future scan of the lumbar region only under light sedation, reducing the time of image acquisition with respect to traditional imaging techniques and cost compared to MRI and scintigraphy.

In this study, the CT examination provided detailed information about bone features in the case of pathological changes in the lumbar spine, adding to the ones revealed by radiographic, ultrasonographic, and scintigraphic evaluations through the years, thus enhancing the knowledge of the most caudal lumbar vertebral abnormalities and limitations of the first-level diagnostic techniques.

## Figures and Tables

**Table 1:** Age (mean and standard deviation) and body weight (median and ranges), number (*n*), and prevalence (%) of sex distribution, breed, and previous use for 40 specimens that underwent post-mortem computed tomographic evaluation of the caudal lumbar region.

Horses (*n* = 40; %)		
Age (years)	16; 6	
Body weight (kg)	500; 400–620	
Sex	Male and geldings (13; 32.5%)	
	Female (27; 67.5%)	
Breed	Warmblood (17; 42.5%)	
	Arab (5; 12.5%)	
	Anglo-Arab (4; 10%)	
	Thoroughbred (4; 10%)	
	Standardbred (3; 7.5%)	
	Others (7; 17.5%)	
Previous use	Athletic use (31; 77.5%)	jumping (17; 42.5%)
		racing (7; 17.5%)
		showing (4; 10%)
		others (3; 7.5%)
	Nonathletic use (9; 22.5%)	

**Table 2:** Number (*n*), prevalence (%), and mean Hounsfield unit (HU) of L(iv)-L(i) lumbar SP contact and fusion for 40 specimens that underwent post-mortem computed tomographic evaluation of the caudal lumbar region. D: dorsal; M: middle; V: ventral; SP: spinous process; L(iv): fourth to the most caudal lumbar vertebra; L(iii): third to the most caudal lumbar vertebra; L(ii): second to the most caudal lumbar vertebra; L(i): the most caudal lumbar vertebra.

	Specimens *n* (%)	L(iv)-L(iii) *n* (%; Mean HU)	L(iii)-L(ii) *n* (%; Mean HU)	L(ii)-L(i) *n* (%; Mean HU)	L(iii)-L(i) *n* (%)	L(iv)-L(ii) *n* (%)
SP contact	21 (54%)	-	12 (57%;327)	4 (19%;318)	4 (19%)	1 (5%)
SP fusion	6 (15%)	V: 1 (17%;625)	D: 2 (33%;467) D and M: 1 (17%;488)	V: 2 (33%;707)	-	

**Table 3:** Number (*n*), prevalence (%), mean bone density expressed as Hounsfield Units (HU), and mean length (cm) of the most caudal lumbar intertransverse joints (ITJs) and degree of osseous pathological changes for 40 specimens that underwent post-mortem computed tomographic evaluation of the caudal lumbar region. L: left; R: right; B: bilaterally; S: symmetry; L(iv): fourth to the most caudal lumbar vertebra; L(iii): third to the most caudal lumbar vertebra; L(ii): second to the most caudal lumbar vertebra; L(i): the most caudal lumbar vertebra.

ITJs	Specimens *n* (%)				Mean HU		Mean Length (cm)		Fusion *n* (%)		
	Only L	Only R	B	S	L	R	L	R	L	R	S
L(iv)-L(iii)	-	1 (2.5%)	-	-	-	665	-	2.4	-	1 (2.5%)	-
L(iii)-L(ii)	4 (10%)	-	19 (47.5%)	19 (83%)	451	397	3.2	3	5 (22%)	5 (26%)	4 (67%)
L(ii)-L(i)	-	-	40 (100%)	40 (100%)	329	294	4.5	4.5	13 (32.5%)	18 (45%)	11 (58%)

**Table 4:** Number (*n*) and prevalence (%) of degree of pathological changes of the L(iv)-L(i) articular processes joints (APJs) for 40 specimens that underwent post-mortem computed tomographic evaluation of the caudal lumbar region. L: left; R: right; B: bilaterally; S: symmetry; L(iv): fourth to the most caudal lumbar vertebra; L(iii): third to the most caudal lumbar vertebra; L(ii): second to the most caudal lumbar vertebra; L(i): the most caudal lumbar vertebra.

APJs	Osseous Pathological Changes *n* (%)			Severity of Osseous Pathological Changes								
				Mild *n* (%)		Moderate *n* (%)		Severe *n* (%)		Ankylosis *n* (%)		
	L	R	B	L	R	L	R	L	R	L	R	S
L(iv)-L(iii)	1 (2.5%)	-	20 (50%)	7 (33%)	6 (30%)	5 (24%)	5 (25%)	6 (29%)	6 (30%)	3 (14%)	3 (15%)	18 (86%)
L(iii)-L(ii)	-	1 (2.5%)	31 (77.5%)	7 (22%)	8 (25%)	12 (37%)	11 (34%)	6 (19%)	6 (19%)	7 (22%)	7 (22%)	30 (91%)
L(ii)-L(i)	-	-	28 (70%)	5 (18%)	5 (16%)	11 (35%)	10 (36%)	8 (26%)	7 (25%)	7 (23%)	6 (21%)	27 (96%)

**Table 5:** Mean bone density (Hounsfield Units) (mean HU) of the cranial and caudal endplate of the vertebral bodies from the cranial L(iv) to the cranial L(i) endplate; number (*n*), prevalence (%), and localization of lumbar spondylosis for 40 specimens which underwent post-mortem computed tomographic evaluation of the caudal lumbar region. L(iv): fourth to the most caudal lumbar vertebra; L(iii): third to the most caudal lumbar vertebra; L(ii): second to the most caudal lumbar vertebra; L(i): the most caudal lumbar vertebra.

Specimens (*n* = 40;%)		Mean HU	Spondylosis *n* (%)
		-	17 (42.5%)
Localization	Ventral	-	5 (29%)
	Ventral and Lateral	-	12 (71%)
	Cranial L(iv)	642	6 (35%)
	Caudal L(iv)	884	10 (59%)
	Cranial L(iii)	636	10 (59%)
	Caudal L(iii)	721	4 (24%)
	Cranial L(ii)	522	7 (41%)
	Caudal L(ii)	565	1 (6%)
	Cranial L(i)	380	-

## Data Availability

The data presented in this study are available on request from the corresponding author.
